# Book review: The ants of Poland with reference to the myrmecofauna of Europe - Fauna Poloniae (New series) vol. 4 By Wojciech Czechowski, Alexander Radchenko, Wiesława Czechowska, Kari  Vepsäläinen

**DOI:** 10.3897/zookeys.247.4369

**Published:** 2012-11-30

**Authors:** Vera Antonova

**Affiliations:** 1 Institute of Biodiversity and Ecosystem Research – Bulgarian Academy of Sciences, 2 Y. Gagarin Str., Sofia, Bulgaria

**Figure:**
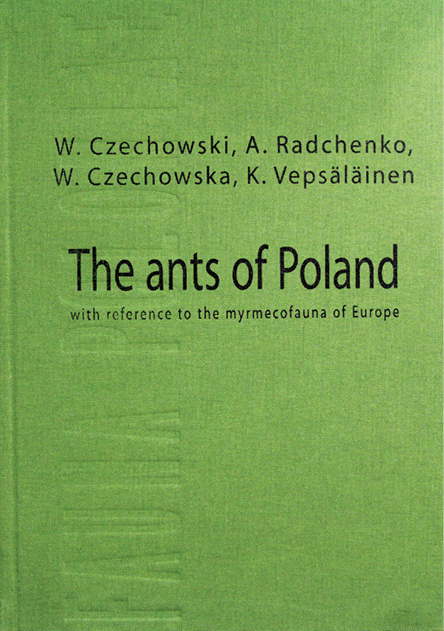


The four authors are well-known in the field of European myrmecology. Research on the myrmecofauna of Poland has a long history. Due to the continuity in the work of myrmecologists of different generations, it seems likely that their good work will be extended successfully into the future. The fruitful work of the late Prof. Bohdan Pisarski has been continued successfully by Wojciech Czechowski and Wiesława Czechowska. They and their students have worked intensively on the Polish myrmecofauna for several decades and they have amassed a great database on every part of Poland. The well-known Palaearctic myrmecologist Aleaxander Radchenko has made outstanding contributions to the knowledge of European myrmecofauna. Kari Vepsäläinen and his team in Finland have had a longstanding collaboration with their Polish colleagues.

The present book is the fourth volume of the new series of Fauna Poloniae. The former monograph “The ants of Poland” (Czechowski et al. 2002) has been considerably updated. It incorporates the latest developments in the taxonomy of ants and has been improved by the inclusion of scanning photographs, detailed drawings of representative ant species by castes, notes on the conservation status of the species, spatial analysis of the Polish ant fauna by regions as well as faunistical, zoogeographical and ecological comparisons of the Polish myrmecofauna with European fauna as a whole and with that of other countries.

The monograph includes the 103 species of ants from 25 genera reported for Poland up until 2010.

The book is in English, separated into 4 chapters:

**Chapter 1:** **Checklist of the ant taxa of Europe.** A checklist for European myrmecofauna is published with nine subfamilies, 57 genera and 613 valid species. The list is in alphabetical order. This is the most recently updated list of the European ants, and shows the recent changes in ant taxonomy.

**Chapter 2:** **Faunistic catalogue of the ants of Poland.** This chapter includes a taxonomic survey of the Polish ant species. For every species there is enclosed: Latin name, taxonomic history, general distribution in the Palaearctic region with map, the distribution in 21 geographical regions in Poland with map and joint table, notes on the biology and conservation status of the species. Drawings of a representative species are given for every genus including worker, queen and male. A list with 17 species excluded from the Polish myrmecofauna is given. The reason for their elimination is noted for each species.

**Chapter 3:** **Characteristics of the Polish myrmecofauna.** The chapteris devoted to analyses of species richness and composition, zoogeographical and ecological composition of the Polish myrmecofauna, with reference to the European one. Among 103 Polish ant species, 97 are outdoor and six are entirely synanthropical species.

Contemporary statistical ecological methods are used for spatial analysis of Polish myrmecofauna by regions and among other 12 European countries (with recent check-lists).

The core of the ant fauna is formed by common species occurring in almost all Polish geographical regions. The richest region is Pieniny Mountains with 64 known species. No significant difference is calculated between the mean numbers of ant species in lowlands and uplands. A Central-European myrmecofauna and species with wide range of ecological tolerance prevail in Poland.

The most abundant zoogeographical element in Polish myrmecofauna is the Euro-Caucasian one followed by Euro-West Siberian and Boreo-Montane elements.

Information about the environmental requirements and habitats is reported for each species. With regards to ecological plasticity, oligotopic ant species are observed to prevail in Poland. Three species are classified as everytopic. In relationship to humidity conditions, mesohygro-xerophilic and mesohygrophilic categories are the richest. In respect to temperature conditions species with moderate requirements prevail. No significant differences are shown among the geographical zones in Poland by zoogeographical and ecological composition.

Comparing the Polish myrmecofauna with the European as a whole, the genera *Formica*, *Lasius* and *Myrmica* are significantly overrepresented. The authors investigated changes in species number and presence-absence of outdoor myrmecofauna among 12 European countries along a North-South gradient with detailed discussions.

**Chapter 4:** **Keys for identification.** In this chapter are included keys for identification of subfamilies, genera and species of ants, separately for workers, queens and males (when they are distinguishable). The keys for subfamilies and genera include all European taxa of these ranks. The keys for species include all ant species known in Poland as well as those present in adjacent countries that are recognized as possibly to be found in Poland in the future. The keys are illustrated with excellent quantity SEM photographs of key exterior body characters.

Ant identification is a difficult and somewhat uncertain task. Myrmecologists and students do need a good key for European myrmecofauna. The recent key for Polish ant species will be a useful reference book for European, especially for Central European, specialists.

The present book devoted to the ant fauna of Poland will be very valuable for the libraries of universities and scientific institutions, and thus, it will be of use to European myrmecologists, entomologists, ecologists, conservation biologists, students and all the people interested in the world of ants.

Price: 95 € (173 × 243 mm, hardcover, 496 pages)

ISBN 978-83-930773-4-2
